# Indoleamine 2,3-dioxygenase expression and activity in patients with hepatitis C virus-induced liver cirrhosis

**DOI:** 10.3892/etm.2014.2146

**Published:** 2014-12-18

**Authors:** KASHIF ASGHAR, M. TAIMOUR ASHIQ, BILAL ZULFIQAR, AMNAH MAHROO, KAENAT NASIR, SHEEBA MURAD

**Affiliations:** Molecular Immunology Research Group, Atta-ur-Rahman School of Applied Biosciences (ASAB), National University of Sciences & Technology (NUST), Islamabad 44000, Pakistan

**Keywords:** indoleamine 2,3-dioxygenase, hepatitis C virus, liver cirrhosis

## Abstract

Indoleamine 2,3-dioxygenase (IDO) is an immunoregulatory enzyme. It plays a key role in various malignancies, infection and autoimmune diseases. IDO induces immunosuppression through the depletion of tryptophan and its downstream metabolites. Hepatitis C virus (HCV) has infected more than 12 million individuals in Pakistan. The aim of the present study was to assess the expression and activity of IDO in HCV-infected patients. The functional enzymatic activity of IDO was measured by colorimetric assay. Serum samples from 100 HCV-infected patients were taken to examine IDO activity and samples from 100 healthy volunteers were used as controls. Liver sections from patients with HCV (n=35) and healthy controls (n=5) were used for immunohistochemical studies. Immunohistochemical analysis revealed that IDO was overexpressed in 28 of 35 (80%) cirrhotic liver samples, whereas 5 of 35 (14.2%) cases presented moderate and 2 of 35 (5.7%) cases presented mild expression of IDO. The enzymatic activity of IDO was significantly higher in the serum samples of HCV-infected patients as compared with those in the control. These data indicate that the expression of IDO correlated with the pathogenesis of disease. In summary, it is suggested that the high expression of IDO in the progressively cirrhotic livers of HCV-infected patients might contribute to the development of hepatocellular carcinoma. IDO may characterize a novel therapeutic target against HCV.

## Introduction

Hepatitis C virus (HCV), a member of family Flaviviridae (1), has infected an estimated 170 million people worldwide and patients have high risk of chronic liver diseases and liver cancer (2–4). The delayed responses of lymphocytes are linked to chronic HCV infection (5). Efforts to improve the understanding of the pathogenesis of the disease and to identify novel therapeutic targets are emergent.

Indoleamine 2,3-dioxygenase (IDO) is a heme-containing, immunosuppressive enzyme which degrades tryptophan into kynurenine (6). Lipopolysaccharide (LPS), interleukin-1 (IL-1) and tumor necrosis factor (TNF) act synergistically with interferon-γ (IFN-γ) to enhance IDO expression *in vitro* (7). IFN-γ is upregulated in the livers of patients with chronic hepatitis C (CHC) infection (8). The overexpression of IDO induces tolerance and immunosuppression, and studies of different models have confirmed that IDO is a potent regulator of adaptive immune responses that has the ability to suppress T-cell proliferation through tryptophan depletion (9–11). IDO activity is inhibited by 1-methyl tryptophan (1-MT) (11). A role of IDO in immune evasion by cancer has been proposed and the inhibition of IDO *in vivo* could be a promising antitumor adjuvant therapy (12).

A high expression of IDO in the liver has been described in humans with chronic infection and HCV clearance has been found to be associated with the normalization of the levels of IDO (13). IDO may suppress T-cell reactivity to viral antigens in CHC infection. A study revealed that in HCV-infected chimpanzees that cleared the infection, the hepatic IDO expression level was normal; however, it was high in those who developed liver cirrhosis (13). In humans, T-regulatory cells are expended for the period of acute HCV infection (14–16), maintained during the chronic stage (14,17–20) and decrease in individuals who recover from HCV (14,18). Fallarino *et al* (21) observed that tryptophan-derived catabolites and tryptophan starvation can transform naïve CD4^+^ CD25^–^ T cells into CD4^+^ CD25^+^ and FoxP3^+^ regulatory T cells. A previous study has shown that mature dendritic cells expand CD4^+^CD25 high regulatory T cells in an IDO-dependent manner (22). IDO-expressing dendritic cells (DCs) enhance the function of Tregs (23). IDO inhibits T-cell responses through tryptophan metabolites that are produced by the kynurenine pathway (24). The blocking of IDO by 1-MT might result in augmented T-cell propagation in DC-T cell co-culture *in vitro* (25), which may be promising new adjuvant therapeutic target for HCV. The authors of the present study hypothesized that IDO may be involved in HCV-induced liver cirrhosis. Thus, i) the functional enzymatic activity of IDO in the serum samples of patients with HCV and controls and; ii) the expression of IDO in control and HCV-infected liver tissues was investigated in the current study.

## Materials and methods

### Patients

A total of 240 individuals were involved in the current study. The IDO enzymatic activity was analyzed in the serum samples of 200 individuals (100 HCV cases and 100 controls). After blood was obtained, the serum samples were immediately isolated and were stored at −20°C and paraffin-embedded blocks were stored at room temperature for one week. Immunohistochemistry was performed on liver sections from HCV-infected patients (n=35) and healthy controls (n=5). This study was conducted with the approval of the institutional review board (IRB) of Atta-ur-Rahman School of Applied Biosciences (ASAB), National University of Sciences & Technology (NUST; Islamabad, Pakistan). Samples were taken for this study with the consent of patients.

### Immunohistochemistry

Following de-paraffinization, the liver sections were subjected to antigen retrieval by 1 min in a pressure cooker in Tris/EDTA buffer pH 8.0 (T9285; Sigma-Aldrich, St. Louis, MO, USA) followed by rapid cooling in running tap water. The sections were then washed with phosphate-buffered saline (PBS) containing 0.1% Tween 20. Sections were soaked in 3% H_2_O_2_ methanol solution for 5 min, followed by 15 min in biotin blocking solution (X0590; Dako Agilent Technologies, Glostrup, Denmark) and washing with Tris-buffered saline (TBS) 3X for 5 min. To prevent non-specific binding, the sections were incubated at room temperature (RT) for 1 h in 20% normal swine serum (S-4000; Vector Laboratories, Burlingame, CA, USA) in TBS. Mouse monoclonal anti-IDO antibody (ab55305; Abcam, Cambridge, UK) was applied at a dilution of (1:100) in normal swine serum at 4°C in a humidified chamber overnight. Following three washes with TBS for 5 min, the secondary antibody peroxidase horse anti-mouse IgG antibody (PI-2000; Vector Laboratories) diluted 1:100 in normal swine serum was applied for 1 h at RT in a humidified chamber. Following three washes with PBS, the slides were developed with a diaminobenzidine (DAB) peroxidase substrate kit (SK-4100 Vector Laboratories), and counterstained with Mayer’s hematoxylin (Dako, Agilent Technologies) and the staining was visualized with a microscope (B-350; Optika Italy, Ponteranica, Italy).

### Colorimetric assay

Kynurenine was measured spectrophotometrically, as previously described (26,27). In brief, following the addition of 50 μl 30% trichloroacetic acid to 100 μl serum sample, the serum samples were vortexed and centrifuged at 10,000 × g for 5 min. Then, 75 μl of the supernatant was added to an equal volume of Ehrlich’s reagent (100 mg *p*-dimethylaminobenzaldehyde and 5 ml glacial acetic acid) in a microtiter plate well (96-well format). Optical density was measured at 492-nm using an ELx800 Absorbance Microplate reader (Dynex Technologies, Chantilly, VA, USA). A standard curve of defined kynurenine concentration (0–100 μM) permitted the analysis of unknown concentrations.

### Statistical analysis

Statistical analysis was carried out using GraphPad Prism 3.0 software (GraphPad Software, San Diego, CA, USA). Unless otherwise stated, graphical data represents the mean value of an experiment performed in triplicate using the Student’s t-test if the level of significance reached P<0.05.

## Results

### Immunohistochemical staining of IDO in liver samples with HCV-induced cirrhosis

**Figure 1:**
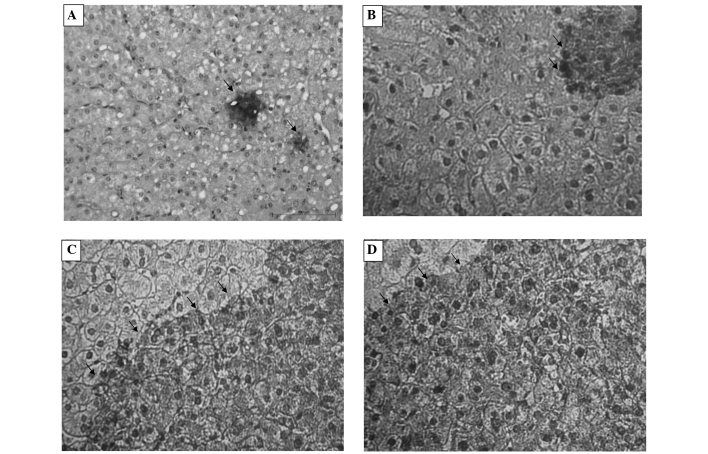
Expression of IDO in HCV infected liver biopsies. The IDO expression was examined in liver tissues taken from healthy controls (n=5) and patients with HCV (n=35) using anti-IDO antibody, as described in material and methods. Minimal expression was observed in hepatocytes of healthy controls (A). Mild expression of IDO reveled in the hepatocytes of HCV patients (B). Moderate expression of IDO reveled in the hepatocytes of HCV patients (C). The high expression of IDO was noticed in hepatocytes (D). Images were captured at ×100 magnification.

In order to observe the exact status of IDO expression *in vivo*, IDO protein expression was investigated by immunohistochemistry. No IDO-positive staining was detected in the sections of normal liver tissues, with the exception of minimal expression in hepatocytes Fig. 1A, whereas in the hepatocytes of the 35 liver sections from HCV-infected patients, mild IDO expression was exhibited in two (5.7%) cases (Fig. 1B), moderate IDO expression was present in five (14.2%) cases (Fig. 1C) and high expression of IDO was observed in 28 (80%) cases. The high expression of IDO may represent a significant role in the pathogenesis of disease.

### IDO activity in patient sera

**Figure 2:**
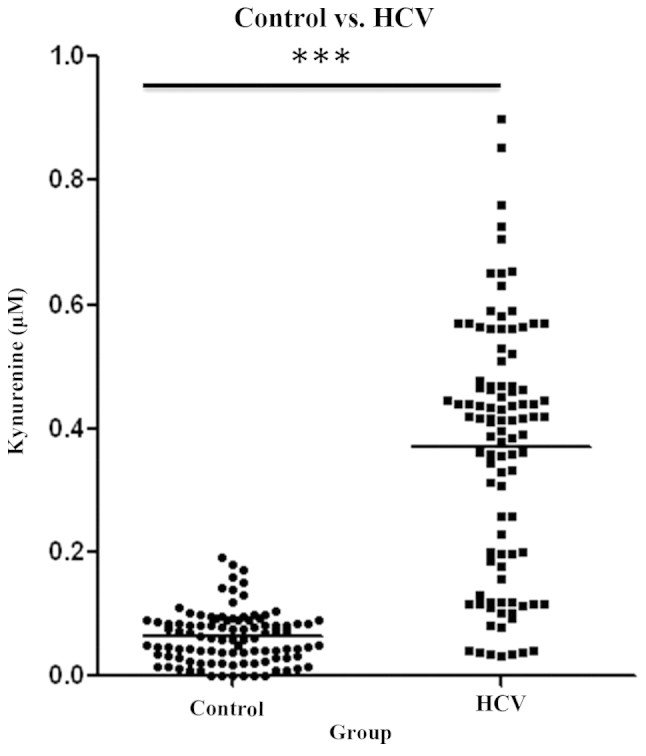
IDO enzymatic activity in serum samples analyzed through a colorimetric assay. A kynurenine detection assay was performed to determine the IDO activity. Kynurenine levels were measured in 200 different samples. IDO, indoleamine 2,3-dioxygenase; HCV, hepatitis C virus.

It was imperative to evaluate IDO enzymatic activity, as there is a documented incongruity between IDO activity and expression, indicating probable post-translational regulation of the enzyme (28,29). The functional enzymatic activity of IDO may be determined by quantifying kynurenine (the first catabolite in the kynurenine pathway). To confirm this approach, a standard curve for quantifying kynurenine was established. Significantly high levels of kynurenine were observed in the HCV-infected patients as compared with those in the control group (Fig. 2).

## Discussion

In the current study, IDO expression was analyzed in liver tissue by immunohistochemistry and the correlation of IDO expression level and functional enzymatic activity was evaluated. To the best of our knowledge, this is the first time that this has been evaluated in Pakistan. The findings demonstrate that IDO expression was detectable in cirrhotic cells. High IDO expression possibly arose due to active inflammation; this is consistent with results obtained by Pan *et a*l (30). The IDO expression levels were significantly higher in the HCV-infected patients as compared with those in the control group. This requires investigation with a broader cohort to establish a definitive correlation.

Numerous studies have confirmed that the clearance of HCV infection is linked to HCV-specific CD4+ T-cell responses (31,32,33). The exact mechanism underlying the failure of certain individuals to resolve HCV infection is poorly understood. The overexpression of IDO induces immunosuppression. IDO has the ability to suppress T-cell proliferation through tryptophan depletion (9–11). It has previously been shown that IDO creates a transitional pathway in dendritic cell maturation leading to the expansion of CD4^+^CD25 high regulatory T cells (22). IDO-expressing DCs enhance the function of Tregs (23) and an augmented number of Tregs at the commencement of infection has been defined as a chronic infection (34). Ino *et al* (35) and Brandacher *et al* (36) have confirmed that high IDO expression levels contribute to the metastasis of endometrial and colorectal cancer. Moreover, the high immunoreactivity of IDO is significantly associated with the frequency of liver metastases (37). The data in the present study indicate that IDO is a crucial player that may contribute to the poor outcome of patients in a manner that remains unknown.

The role of IDO in HCV is only just beginning to be studied in detail. The current study proposes that IDO mediates the immune escape employed by HCV in chronic patients and, therefore, more studies into the exact mechanism by which HCV signaling leads to the upregulation of IDO are warranted. These data support the hypothesis that an immunosuppressive environment created by IDO may lead patients with chronic HCV infection progressively toward liver cirrhosis. IDO has the potential to become a useful marker for HCV-induced liver cirrhosis. Thus, the inhibition of IDO activity may contribute to the application of adjuvant therapy intervention for HCV.
